# Ambient air pollutants and outpatient visits for conjunctivitis in Hefei, China: a time-series study of hospital-based data during 2014–2023

**DOI:** 10.3389/fpubh.2026.1926405

**Published:** 2026-09-23

**Authors:** Xuan Zhang, Yunyi Jia, Di Tian, Xiaojie Zou, Deshen Mao, Fangyuan Zhang, Jian Cheng, Zhengyu Gu

**Affiliations:** 1 Department of Ophthalmology, The First Affiliated Hospital of Anhui Medical University, Hefei, China; 2 First Clinical Medical College, Anhui Medical University, Hefei, China; 3 Department of Organization, The First Affiliated Hospital of Anhui Medical University, Hefei, Anhui, China; 4 School of Public Health, Anhui Medical University, Hefei, Anhui, China; 5 Department of Epidemiology and Biostatistics, School of Public Health, Anhui Medical University, Hefei, China; 6 Huaibei People’s Hospital, Huaibei, China, Huaibei Municipal Key Laboratory of Visual Health, Huaibei, China

**Keywords:** air pollution, conjunctivitis, environmental epidemiology, gaseous pollutants, outpatient visits, particulate matter, time-series study

## Abstract

**Background:**

Ambient air pollutants can directly act on the conjunctiva, triggering or exacerbating conjunctivitis. Nevertheless, evidence regarding the effects of common air pollutants on conjunctiva remains limited.

**Methods:**

We collected 73,043 outpatient records of conjunctivitis visits to hospitals in Hefei from 2014 to 2023. A time-series study was adopted to investigate the short-term associations between five common air pollutants [i.e., aerodynamic diameter ≤2.5 μm (PM₂.₅), particulate matter with an aerodynamic diameter ≤10 μm (PM₁₀), ozone (O₃), nitrogen dioxide (NO₂), and sulfur dioxide (SO₂) and the risk of outpatient visits for conjunctivitis]. Furthermore, we explored the potential effect modification by gender, age, season, and the COVID-19 pandemic.

**Results:**

Exposures to all five ambient air pollutants were positively associated with elevated daily outpatient visit risks for conjunctivitis, with the strongest single-day effects generally observed on the concurrent day of exposure (lag 0), while cumulative analyses indicated pollutant-specific short-term lag patterns. The magnitude of associations varied across pollutants; SO₂ showed the largest lag-0 effect estimate, followed by NO₂. Stratified analyses further revealed that season, gender, age, and study period modified the pollutant-conjunctivitis associations to varying degrees.

**Conclusion:**

This study found that short-term exposure to PM_2.5_, PM₁₀, O₃, NO₂, and SO₂ was associated with increased outpatient visits for conjunctivitis, with evident subgroup heterogeneity. These findings may have implications for clinical practice and public health in the prevention of eye diseases against air pollution.

## 1 Introduction

Conjunctivitis is a common ocular surface disorder characterized by conjunctival hyperemia, tearing, ocular itching, foreign-body sensation, discharge, and ocular irritation (1, 2). Given the substantial and persistent health burden of ambient air pollution in recent years (3, 4), increasing attention has been directed toward its potential adverse effects on ocular surface health, including conjunctivitis. Unlike many internal organs, the ocular surface is directly exposed to the external environment; therefore, airborne particles and gaseous pollutants may interact directly with the tear film, conjunctival epithelium, and ocular surface immune microenvironment (5–7).

Ambient air pollutants may affect the ocular surface through multiple biological pathways. Particulate matter can deposit on the ocular surface, disrupt tear-film stability, and induce oxidative stress and local inflammation (8). Gaseous pollutants, including ozone, nitrogen dioxide, and sulfur dioxide, may act as mucosal irritants and oxidizing agents, thereby impairing epithelial barrier function and promoting inflammatory responses (9). These processes may trigger ocular discomfort and increase the likelihood of healthcare-seeking for conjunctivitis.

Growing epidemiological evidence has explored the associations between ambient air pollution and conjunctivitis. Previous studies have examined individual pollutants or selected pollutant mixtures; however, direct comparisons across multiple common pollutants within a unified modelling framework remain limited (10–14). Given the substantial discrepancies in emission sources, atmospheric transport behaviors, chemical properties, and biological effects among fine particulate matter (PM_2.5_, aerodynamic diameter≤2.5 μm), inhalable particulate matter (PM₁₀, aerodynamic diameter≤10 μm), ozone (O₃), nitrogen dioxide (NO₂), and sulfur dioxide (SO₂), this study incorporated these five prevalent ambient air pollutants to evaluate their short-term associations with outpatient visits for conjunctivitis. In addition, stratified analyses were conducted across subgroups of age, gender, season, and study period to assess their potential effect modification.

## 2 Materials and methods

### 2.1 Study population and clinical data

This study was conducted in Hefei, the capital city of Anhui Province in eastern China. According to the Hefei Statistical Yearbook 2024, Hefei covers an area of 11,445 km^2^ and had a permanent resident population of 9.853 million in 2023. As a rapidly urbanizing city, Hefei has experienced substantial population growth, urban expansion, increasing traffic volume, and industrial development, which may contribute to complex multi-pollutant exposure patterns. This city is also located in a climatic transition zone between the temperate and subtropical regions and has distinct seasonal variation, providing a suitable setting to evaluate both the short-term effects of ambient air pollution on conjunctivitis outpatient visits and potential seasonal heterogeneity in pollution-related ocular health risks.

Outpatient visit records were obtained from the First Affiliated Hospital of Anhui Medical University and the Second Affiliated Hospital of Anhui Medical University in Hefei, China, covering the period from 2014 to 2023. A total of 73,043 conjunctivitis-related outpatient visits were identified using ICD-10 diagnostic codes H10.0, H10.1, H10.2, H10.3, H10.4, and H10.9. The analysis was conducted at the visit level. Because repeated visits by the same patient could not be reliably identified in the available records, patient-level deduplication was not performed. All records were routinely collected from hospital administrative systems and core variables included visit date, gender, age, and diagnostic code. These fields provided the basis for the daily time-series analysis, exposure-response assessment, and stratified analyses. This retrospective study used outpatient records generated during routine clinical care between 2014 and 2023. The study protocol was reviewed and approved in 2025 by the Ethics Committee of the First Affiliated Hospital of Anhui Medical University (approval No. PJ 2025-02-20).

### 2.2 Air pollution and meteorological data

Daily concentrations of PM₂.₅, PM₁₀, O₃, NO₂, and SO₂ were obtained from the China High Air Pollutants (CHAP) dataset. City-level exposure was calculated by averaging all valid CHAP grid cells within the administrative area of Hefei. The spatial resolution was 1 km × 1 km for PM₂.₅, PM₁₀, and O₃ throughout the study period; for NO₂ and SO₂, it was 10 km × 10 km before 2019 and 1 km × 1 km from 2019 onward. PM₂.₅, PM₁₀, NO₂, and SO₂ were represented by daily mean concentrations, whereas O₃ was represented by the maximum daily 8-h average (MDA8). No missing pollutant data were identified during the study period. The CHAP dataset integrates multiple data sources and modelling approaches to characterize spatiotemporal variations in ambient air pollution (15–18). Recent independent studies have further evaluated the accuracy and multiscale applicability of CHAP-derived exposure estimates in China (19–22). Daily mean temperature and daily relative humidity were derived from the ERA5-Land reanalysis dataset, which provides hourly meteorological variables at an approximate spatial resolution of 9 km.

### 2.3 Statistical analysis

A generalized additive model combined with a distributed lag non-linear model was used to estimate the short-term associations between each air pollutant and daily outpatient visits for conjunctivitis (11, 23, 24). For each pollutant, alternative exposure–response specifications were compared using the Akaike information criterion (AIC), and the linear exposure–response function was selected for the cross-basis construction to improve model parsimony, interpretability, and comparability across pollutants. The lag-response dimension was modelled using a natural cubic spline with 3 degrees of freedom, and the maximum lag was set to 7 days to capture short-term and delayed effects. Daily conjunctivitis outpatient visit counts were assumed to follow a Poisson distribution with a log link function. The model was specified as follows:

$$\begin{array}{l} \log[E(Y_{t})]=a+\mathrm{cb}(P_{t},\mathrm{lag}=0-7)+\mathrm{ns}(\mathrm{Tem}p_{t},\mathrm{df}=3)\\ +\mathrm{ns}({\mathrm{RH}}_{t},\mathrm{df}=3)+\mathrm{ns}(\text{Time},\mathrm{df}=7/\text{year})+\mathrm{\gamma DO}W_{t}+\delta {\text{Holiday}}_{t} \end{array}$$

Where *Y_t_* denotes the number of conjunctivitis outpatient visits on day t; *μₜ = E(Yₜ)* is the expected daily visit count, α is the intercept, *Pₜ* is the daily concentration of a given pollutant, cb(·) denotes the cross-basis function, *ns(·)* denotes a natural cubic spline, *Tempₜ* and *RHₜ* denote daily mean temperature and relative humidity, respectively, *Timeₜ* represents calendar time, *DOWₜ* denotes day of the week, and *Holidayₜ* denotes public holidays. Long-term and seasonal trends were controlled using a spline function for calendar time. The robustness of these specifications was further evaluated by varying the df for temperature and relative humidity to 2, 4, and 5 and the df for calendar time to 6, 8, and 9 per year, while keeping all other model settings unchanged.

Effect estimates were expressed as relative risks (RRs) with 95% confidence intervals per 1 μg/m^3^ increase in pollutant concentration for single-day lags from lag 0 to lag 7 and cumulative lag periods of lag 0–1, lag 0–3, and lag 0–7 (25, 26). Given the correlations among pollutants, selected two-pollutant models were additionally fitted as sensitivity analyses to assess potential co-pollutant confounding. NO₂ was separately modeled with PM₂.₅, PM₁₀, and SO₂, using the same lag structure and covariate adjustment as in the primary models.

Stratified analyses were performed to assess whether the effects of air-pollution exposure on daily conjunctivitis outpatient visits varied across population and temporal subgroups. Gender- and age-stratified analyses were conducted to identify potential susceptible populations (27), with gender classified as male or female and age grouped into ≤18 years, 18–65 years, and >65 years. Seasonal stratification was further performed because air-pollution levels, meteorological conditions, outdoor activity patterns, and conjunctivitis visits may vary by season (28). Seasons were defined as spring (March–May), summer (June–August), autumn (September–November), and winter (December–February). The study period was primarily classified into the pre-pandemic period (2014–2019) and the pandemic period (2020–2023). To assess potential heterogeneity within 2020–2023, an additional sensitivity analysis further divided this period into the pandemic-control period (2020–2022) and the post-control transition period (2023) (29, 30). The Z-test was used to test the subgroup difference.

## 3 Results

During 2014–2023, a total of 73,043 conjunctivitis outpatient visits were recorded. The mean number of daily visits was 20.0, with a median of 18 visits and an interquartile range of 6–33 visits. Females accounted for 55.9% of visits. The age distribution showed that 10.8% were patients aged ≤18 years, 71.8% were those aged 18–65 years, and 17.4% were those aged >65 years. Descriptive statistics for ambient air pollutants and meteorological variables are presented in Table 1 and Figure 1. The mean concentrations were 45.72 μg/m^3^ for PM_2.5_, 76.07 μg/m^3^ for PM_10_, 96.99 μg/m^3^ for O_3_, 30.00 μg/m^3^ for NO_2_, and 13.01 μg/m^3^ for SO_2_. The mean daily temperature was 16.71 °C, and the mean daily relative humidity was 74.62%. Spearman correlation analysis showed that the five air pollutants were significantly correlated with each other (Table 2).

**Table 1:** Distribution of ambient air pollutants and meteorological variables during 2014–2023.

Variable	Min	Mean	SD	Median	P_25_	P_75_	Max
PM_2.5_	4.84	45.72	27.42	39.17	26.01	58.15	232.60
PM_10_	9.62	76.07	39.37	68.05	47.78	96.76	560.70
NO_2_	8.04	30.00	12.74	27.63	21.09	36.78	98.36
O_3_	11.75	96.99	39.96	88.27	65.80	125.99	223.18
SO_2_	4.55	13.01	6.13	11.03	8.17	16.61	43.11
Temp(°C)	−9.29	16.71	8.85	17.57	8.91	24.34	33.52
RH (%)	25.88	74.62	11.41	75.67	67.23	83.02	99.99

SD, standard deviation; P_25_, 25th percentile; P_75_, 75th percentile; PM_2.5,_ fine particulate matter; PM_10,_ inhalable particulate matter; NO_2,_ nitrogen dioxide; O_3,_ozone; SO_2,_ sulfur dioxide; Temp, daily mean temperature; RH, relative humidity.

**Figure 1:**
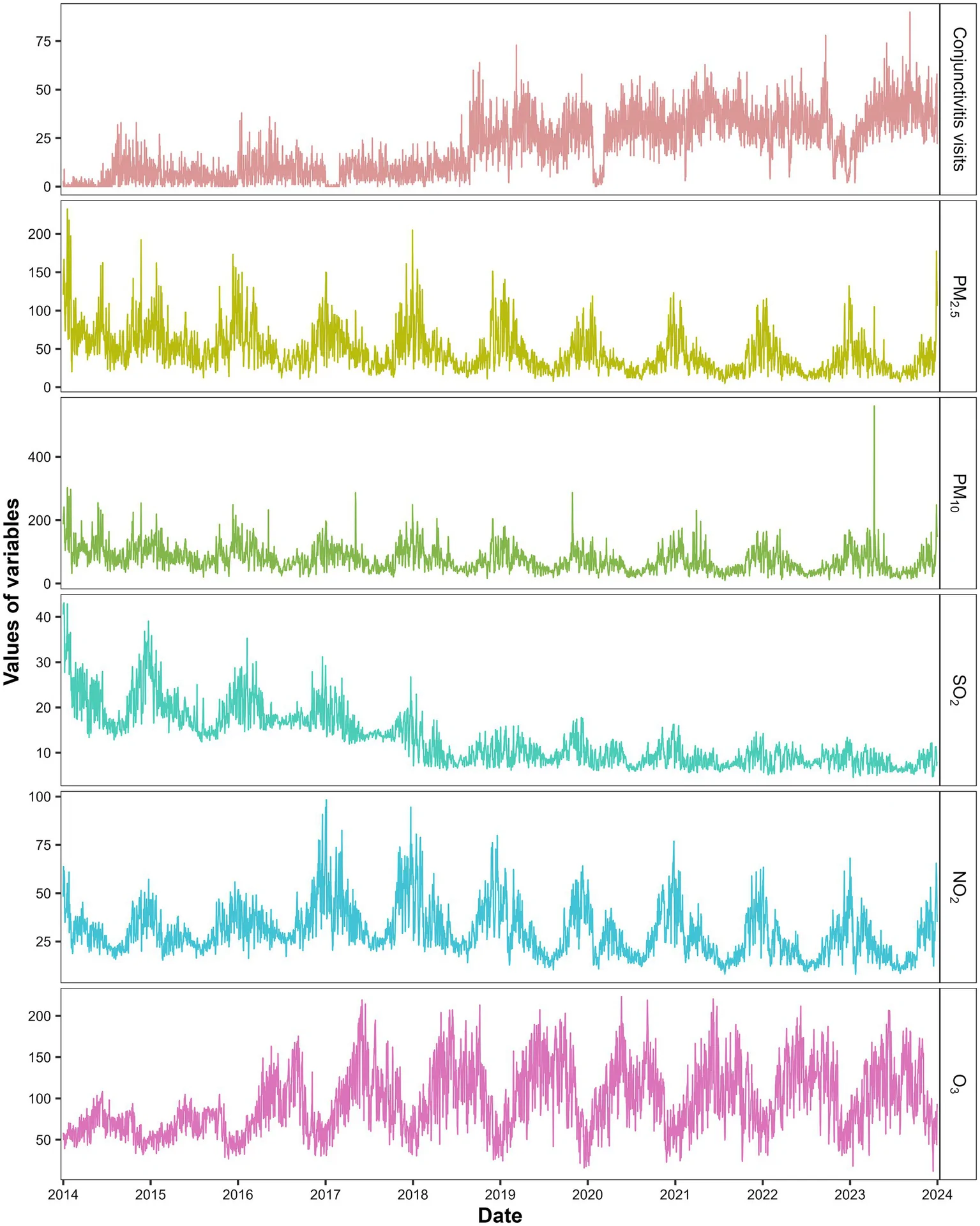
Time-series distribution of daily conjunctivitis visits and ambient air pollutants in Hefei, China, 2014–2023.

**Table 2:** Correlation matrix of daily air pollutant concentrations.

Pollutant	PM_2.5_	PM_10_	O_3_	NO_2_	SO_2_
PM_2.5_	1				
PM_10_	0.925^a^	1			
O_3_	−0.357^a^	−0.211^a^	1		
NO_2_	0.726^a^	0.733^a^	−0.290^a^	1	
SO_2_	0.628^a^	0.632^a^	−0.272^a^	0.567^a^	1

^a^*P* < 0.05.

The single-lag associations between ambient air pollutants and daily conjunctivitis outpatient visits are shown in Figure 2. All five pollutants were associated with conjunctivitis outpatient visits, and the highest risk estimates were consistently observed on the current day of exposure (lag 0), indicating an acute effect. At lag 0, all five pollutants showed statistically significant positive associations with conjunctivitis outpatient visits, with the largest effect observed for SO₂ (RR = 1.0123, 95%CI: 1.0095–1.0151), followed by NO₂ (RR = 1.0047, 95%CI: 1.0039–1.0055), PM₁₀ (RR = 1.0007, 95%CI: 1.0005–1.0008), PM₂.₅ (RR = 1.0006, 95%CI: 1.0003–1.0009), and O₃ (RR = 1.0005, 95%CI: 1.0003–1.0006). Cumulative lag analyses showed positive associations for PM₂.₅ and PM₁₀ at lag 0–1 and lag 0–3, while SO₂ and NO₂ remained positively associated through lag 0–7. O₃ was positively associated at lag 0–1 but showed an inverse association at lag 0–7. At lag 0–7, the RRs for SO₂ and NO₂ were 1.0062 (95%CI: 1.0016–1.0108) and 1.0088 (95%CI: 1.0077–1.0100), respectively. Sensitivity analyses using alternative df specifications produced consistent results, supporting the robustness of the main findings (Supplementary Table S2). In two-pollutant sensitivity analyses, the PM₂.₅ association was attenuated after adjustment for PM₁₀ or NO₂, whereas PM₁₀ and NO₂ remained positively associated at lag 0 after co-pollutant adjustment (Supplementary Table S5).

**Figure 2:**
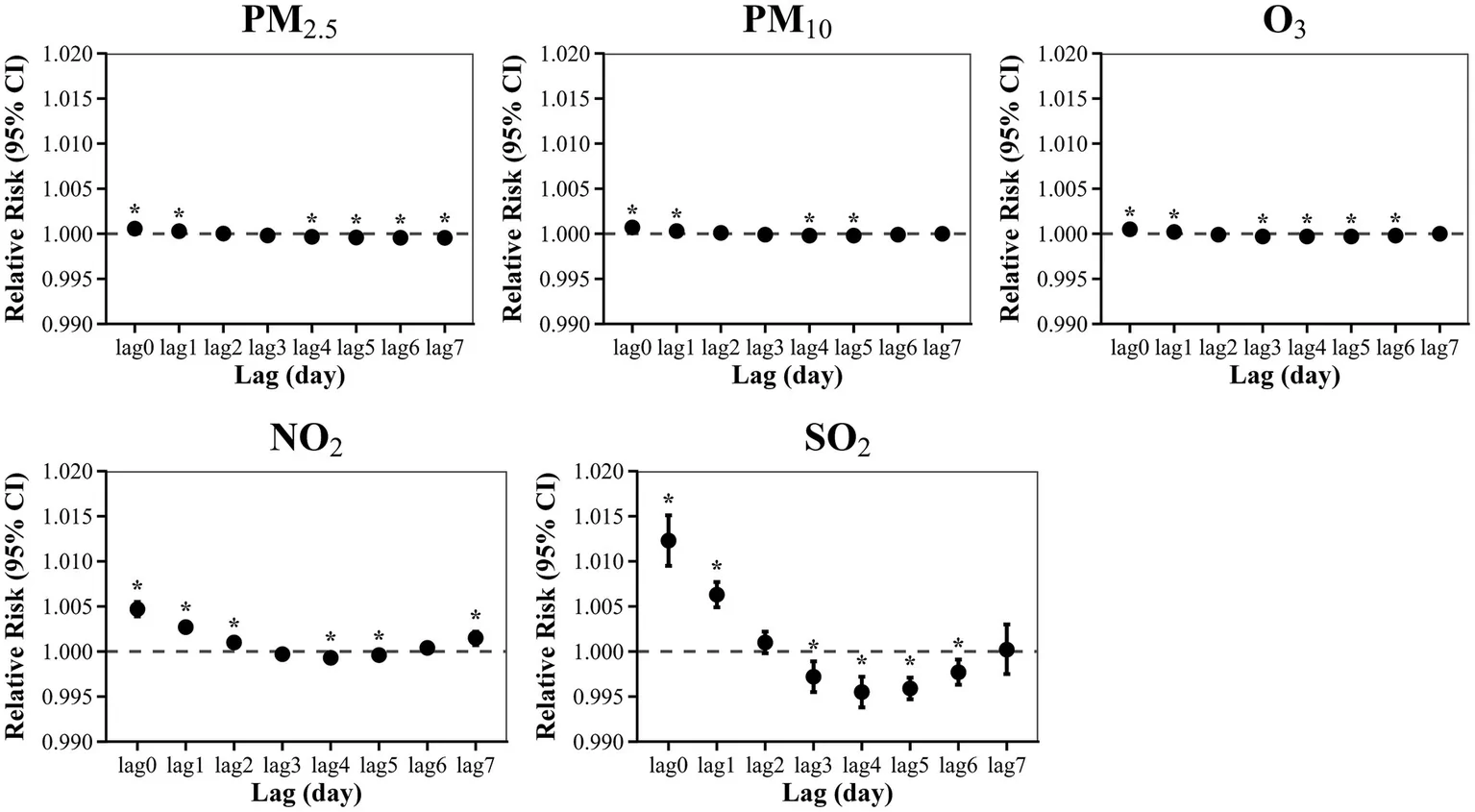
Single-day lag effects of five ambient air pollutants on conjunctivitis outpatient visits.

Exploratory stratified analyses suggested variation in the associations between ambient air pollutants and daily conjunctivitis outpatient visits across seasons, gender, age groups, and study periods(Figure 3). Seasonally, SO_2_ showed significant positive lag-0 associations in spring, autumn, and winter, while NO₂ showed its strongest association in winter (RR = 1.0070, 95%CI: 1.0058–1.0081). O₃ was positively associated in summer and autumn but inversely associated in winter. By gender, SO₂ showed the largest lag-0 estimates in both males (RR = 1.0117, 95%CI: 1.0075–1.0159) and females (RR = 1.0128, 95%CI: 1.0091–1.0166). By age, the largest estimates were also observed for SO₂ in adults aged 18–65 years (RR = 1.0134, 95%CI: 1.0101–1.0167) and >65 years (RR = 1.0123, 95%CI: 1.0052–1.0194). In the study-period analysis, SO_2_ showed the largest lag-0 estimates in both the pre-pandemic period (RR = 1.0158, 95%CI: 1.0118–1.0197) and the pandemic period (RR = 1.0163, 95%CI: 1.011–1.0208), with comparable effect sizes across the two periods (Figure 3). The PM_2.5_ association was significant before the pandemic (RR = 1.0007, 95%CI: 1.0002–1.0012) but was attenuated and no longer statistically significant during 2020–2023 (RR = 1.0004, 95%CI: 0.999–1.0009), whereas the O_3_ association remained relatively stable across the two periods. The cumulative lag associations for all five pollutants are summarized in Figure 4.

**Figure 3:**
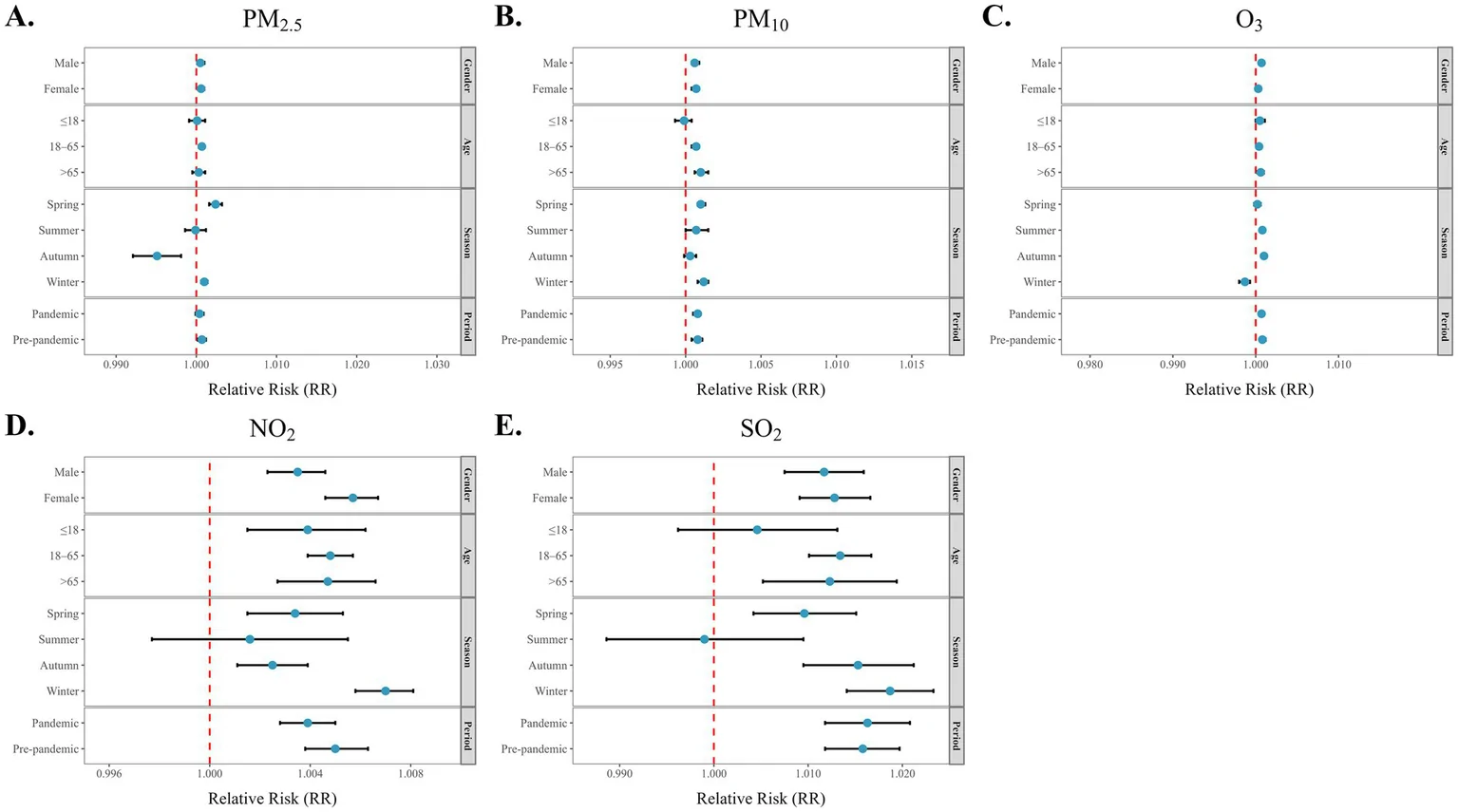
Subgroup associations between ambient air pollutants and daily conjunctivitis outpatient visits across sex, age, season, and study period: **(A)** PM_₂.₅_; **(B)** PM₁₀; **(C)** O_₃_; **(D)** NO_₂_; and **(E)** SO_₂_.

**Figure 4:**
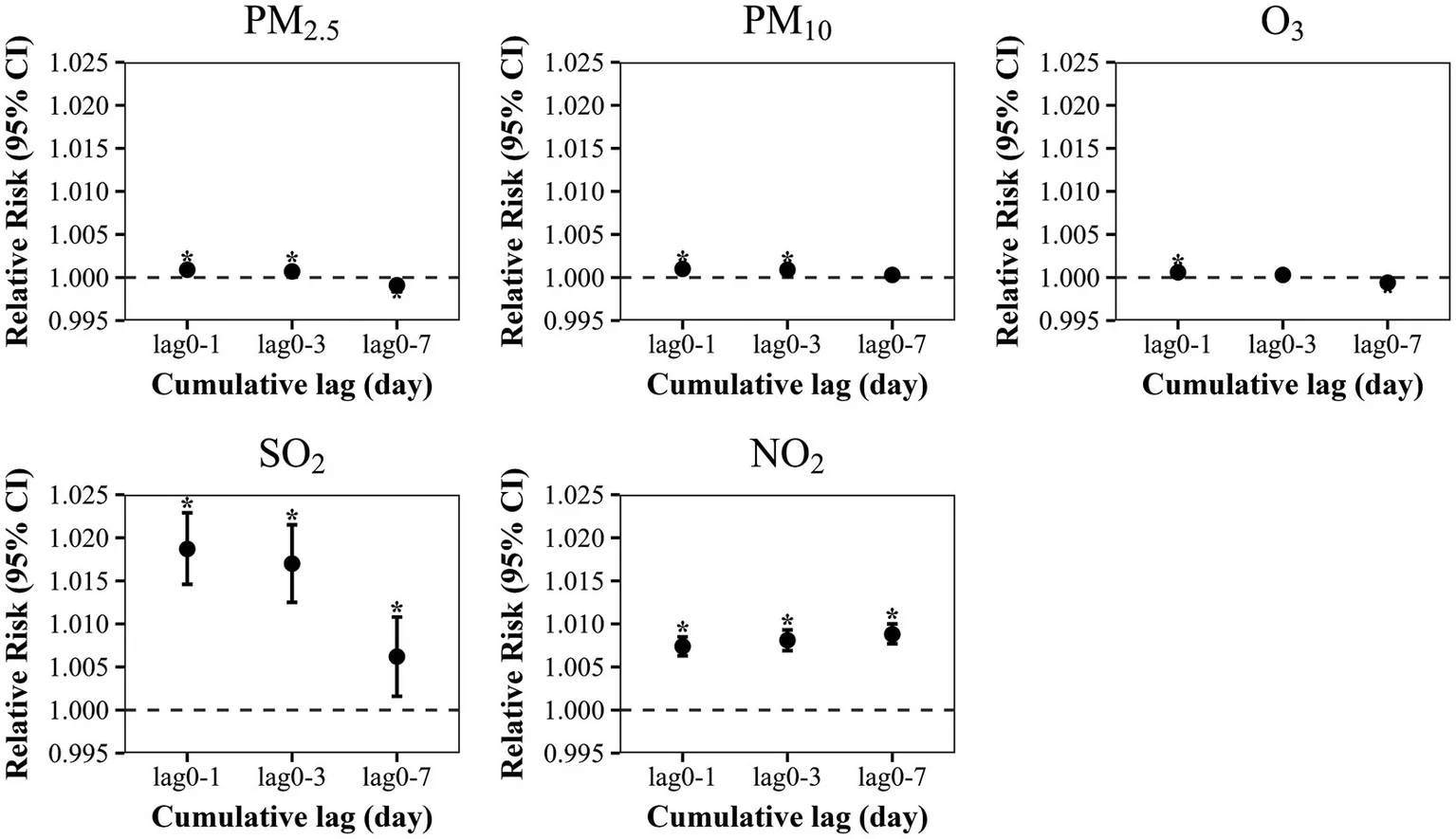
Cumulative lag associations between ambient air pollutants and conjunctivitis outpatient visits.

## 4 Discussion

In this time-series study of conjunctivitis outpatient visits in Hefei from 2014 to 2023, short-term exposure to ambient air pollutants was associated with elevated risk of conjunctivitis consultation. The strongest single-day effects were generally observed on the day of exposure (lag 0), whereas cumulative analyses indicated that short-term associations persisted over subsequent days for several pollutants, particularly SO₂ and NO₂. Exploratory subgroup analyses suggested variation in associations across gender, age, season, and study periods.

Our findings are broadly consistent with previous epidemiological studies linking ambient air pollution to conjunctivitis. In Taiwan, Chang et al. reported that PM₁₀, NO₂, SO₂, and O₃ were associated with increased outpatient visits for nonspecific conjunctivitis, with no evident lag effects (31); besides, the strongest associations for all pollutants also occurred on the day of exposure. In Hangzhou, Fu et al. observed positive same-day associations between particulate pollutants and conjunctivitis outpatient visits, reporting ORs of 1.0092 for PM₂.₅ and 1.0068 for PM₁₀ per 10 μg/m^3^ increase (32), which supports the acute ocular response observed in our study. Moreover, Lu et al. confirmed these associations in a multicity study in China (33), showing positive current day’s associations for PM₂.₅, PM₁₀, NO₂, SO₂, and O₃, with the largest estimate observed for NO₂ (OR = 1.012, 95% CI: 1.005–1.020) per 10 μg/m^3^ increase. Consistent with these findings, our single-pollutant models also showed positive lag-0 associations for all five pollutants; however, given the substantial correlations among co-pollutants, differences in the magnitude of these estimates should not be interpreted as evidence of independent pollutant-specific effects. Although the effect units and modelling approaches differed across studies, the consistent finding of immediate or short-lag associations suggests that conjunctivitis visits may respond rapidly to short-term fluctuations in ambient air pollution.

The observed associations are biologically plausible. The ocular surface is directly exposed to the external environment, allowing particulate matter and gaseous pollutants to interact with the tear film, conjunctival epithelium, and local immune microenvironment (34). Gaseous pollutants such as NO₂ and SO₂ may act as acute mucosal irritants and promote oxidative stress, epithelial barrier disruption, tear-film instability, and local inflammatory responses (6, 9). Particulate matter may deposit on the ocular surface, causing mechanical irritation, oxidative injury, and inflammatory activation (7, 35, 36). Experimental evidence from human conjunctival epithelial cell models also supports this interpretation. Diesel exhaust particles were shown to upregulate inflammatory mediators such as ICAM-1 and IL-6 in primary cultured human conjunctival epithelium (37, 38), and to induce oxidative stress, mucin alterations, and IL-6-mediated inflammatory responses in human conjunctival epithelial cells (39). Taken together, previous epidemiological and experimental findings support our observation that short-term increases in ambient air pollutants may trigger acute ocular surface irritation and inflammation, thereby increasing conjunctivitis-related healthcare visits.

Subgroup analyses further suggested that the associations between air pollution and conjunctivitis varied across season, gender, age, and study periods. Stronger effects of NO_2_ and SO_2_ in the cooler seasons may partly be related to increased combustion emissions, poorer atmospheric dispersion, lower temperature and humidity, and greater ocular surface vulnerability under cold or dry conditions (8, 40, 41). By contrast, O₃ showed a different seasonal pattern, with more evident positive associations during the warm season. This may reflect enhanced photochemical O₃ formation under higher temperatures and stronger solar radiation, together with increased outdoor activity and direct ocular exposure during summer (42). As a strong oxidant, O₃ can induce oxidative stress, epithelial injury, and local inflammatory responses on the ocular surface (43, 44). Recent epidemiological studies have also reported stronger O₃-related associations during summer or under higher-temperature conditions (45, 46). Spring allergens may act as a co-exposure but are unlikely to fully explain this warm-season pattern. The stronger associations observed among females may partly reflect gender-related differences in ocular surface physiology, tear-film characteristics, hormonal regulation, immune responses, cosmetic use, contact lens use, and healthcare-seeking behavior (47–50). The stronger associations observed among adults and older adults for certain pollutants may be related to higher outdoor or occupational exposure, cumulative environmental exposure, age-related ocular surface changes, and reduced epithelial repair capacity (51, 52). In addition, SO₂ showed the largest numerical effect estimates in both the pre-pandemic and pandemic periods, whereas the PM₂.₅ association was attenuated during the pandemic period. These temporal differences may partly reflect changes in pollutant concentrations, population mobility, exposure-related behaviors, and healthcare utilization during the COVID-19 period (53–55).

This study has several strengths. First, the 10-year time-series dataset provided a relatively large and continuous source of conjunctivitis outpatient records in a rapidly urbanizing Chinese city. Second, the use of a unified modelling framework allowed direct comparison of results across five major air pollutants; in addition, stratified analyses helped identify potentially susceptible subgroups and high-risk exposure windows. Several limitations should also be acknowledged. First, city-level CHAP concentrations were used as exposure indicators, and no independent validation against ground-based monitoring measurements in Hefei was performed. Therefore, spatial variation in individual exposure may not have been fully captured, and some exposure measurement error or misclassification cannot be excluded. Second, the outcome included several conjunctivitis subtypes with potentially different etiologies. In addition, patient-level deduplication was not feasible, and repeated or follow-up visits may therefore have been counted as separate encounters, resulting in outcome heterogeneity and potential misclassification. Third, although major meteorological and temporal confounders were adjusted for, residual confounding from factors such as pollen, ultraviolet radiation, indoor air pollution, and individual behaviors cannot be excluded. Fourth, the large number of pollutant-subgroup comparisons may have increased the probability of chance findings and the risk of Type I error. Although Bonferroni correction was applied to pairwise comparisons for subgroup variables with more than two categories, the subgroup analyses remained exploratory and should be interpreted as hypothesis-generating rather than confirmatory.

## 5 Conclusion

In summary, this study evaluated the associations between five ambient air pollutants (PM₂.₅, PM₁₀, O₃, NO₂, and SO₂) and conjunctivitis outpatient visits. The results demonstrated that all pollutants were linked to elevated risk of conjunctivitis consultation, and the strongest single-day effects were generally observed on the concurrent exposure day (lag 0), while cumulative analyses indicated persistent short-term associations for several pollutants, particularly NO₂. Seasonal heterogeneity was also observed: the effect of PM₂.₅ was more pronounced in spring, O₃ showed stronger positive associations in warm seasons, and NO₂ had a more evident impact in winter. These findings provide important epidemiological evidence for developing targeted air-pollution prevention strategies and protecting ocular health.

## Data Availability

The raw data supporting the conclusions of this article will be made available by the authors, without undue reservation.
