# Supplementary material for: Ambient air pollutants and outpatient visits for conjunctivitis in Hefei, China: a time-series study of hospital-based data during 2014–2023
Source: Front Public Health. 2026 Sep 23;14:1926405. doi: 10.3389/fpubh.2026.1926405 (PMC13642795; doi:10.3389/fpubh.2026.1926405)
Supplement: Supplementary file 1 [file Table_1.pdf]

Supplementary Material

Ambient air pollutants and outpatient visits for conjunctivitis in Hefei, China: a time-series study of hospital-based data during 2014–2023

Table S1. Sensitivity Analysis: RR Estimates Rescaled to Pollutant-Specific P_{25} Increments

Pollutant	P25 (μg/m³)	RR per 1 μg/m³ (95%CI)	RR per P_{25} increment (95%CI)
$PM_{2.5}$	26.01	1.0006 (1.0003-1.0009)	1.0150 (1.0065-1.0234)
PM_{10}	47.78	1.0007 (1.0005-1.0008)	1.0340 (1.0242-1.0389)
O_3	65.80	1.0005 (1.0003-1.0006)	1.0334 (1.0199-1.0403)
NO_2	21.09	1.0047 (1.0039-1.0055)	1.1039 (1.0856-1.1226)
SO_2	8.17	1.0123 (1.0095-1.0151)	1.1050 (1.0803-1.1303)

Table S2. Sensitivity analyses and effect estimates.

	Temperature			Relative Humidity			Time		
	df=2	df=4	df=5	df=2	df=4	df=5	df=6/year	df=8/year	df=9/year
$PM_{2.5}$	1.004 (1.001-1.007)	1.006 (1.003-1.009)	1.006 (1.002-1.009)	1.006 (1.003-1.007)	1.006 (1.003-1.009)	1.006 (1.003-1.009)	1.006 (1.002-1.009)	1.006 (1.002-1.009)	1.006 (1.002-1.009)
PM_{10}	1.006 (1.004-1.008)	1.007 (1.005-1.009)	1.007 (1.005-1.009)	1.007 (1.005-1.008)	1.007 (1.005-1.008)	1.007 (1.005-1.008)	1.007 (1.005-1.008)	1.006 (1.004-1.008)	1.004 (1.004-1.008)
O_3	1.006 (1.004-1.007)	1.005 (1.003-1.007)	1.005 (1.003-1.007)	1.005 (1.003-1.006)	1.006 (1.004-1.007)	1.005(1.004-1.007)	1.005 (1.003-1.006)	1.004 (1.003-1.006)	1.005 (1.003-1.006)

SO_2	1.0121 (1.0094-1.0149)	1.0127(1.0099-1.0155)	1.0127(1.0098-1.0155)	1.0123 (1.0095-1.0151)	1.0123 (1.0094-1.0154)	1.0124 (1.00095-1.0155)	1.0129 (1.00101-1.0157)	1.0116(1.0088-1.0144)	1.0120 (1.0092-1.0148)
NO_2	1.0042 (1.0034-1.0050)	1.0047(1.0040-1.0055)	1.0047 (1.040-1.0055)	1.0047(1.0039-1.0055)	1.0047 (1.0040-1.0055)	1.0047(1.0041-1.0056)	1.0049(1.0039-1.005)	1.0046 (1.0039-1.0054)	1.0046(1.0039-1.0054)

Table S3. Significant between-subgroup differences in pollutant–conjunctivitis associations based on Z-tests

Subgroup	Pollutant	Significant comparison(s)	Bonferroni-adjusted P
Gender	O_3	Male vs Female	0.0143
	NO_2	Male vs Female	0.0056
Age	PM_{10}	≤18 vs 18-65 years	0.0284
	PM_{10}	≤18 vs >65 years	0.0073
Season	$PM_{2.5}$	Spring vs Summer	0.0080
	$PM_{2.5}$	Spring vs Autumn	<0.001
	$PM_{2.5}$	Spring vs Winter	0.0129
	$PM_{2.5}$	Summer vs Autumn	0.0244
	$PM_{2.5}$	Autumn vs Winter	<0.001
	PM_{10}	Spring vs Autumn	0.0218
	PM_{10}	Autumn vs Winter	0.0054
	O_3	Spring vs Autumn	0.0103
	O_3	Spring vs Winter	<0.001
	O_3	Summer vs Winter	<0.001
	O_3	Autumn vs Winter	<0.001
	SO_2	Summer vs Autumn	0.0474
	SO_2	Summer vs Winter	0.0047
	NO_2	Spring vs Winter	0.0090

	NO_2	Autumn vs Winter	<0.001

Table S4. Sensitivity analysis of lag-0 associations between ambient air pollutants and conjunctivitis outpatient visits using an alternative three-period classification.

Pollutant	Primary analysis		Sensitivity analysis	
	2014-2019	2020-2023	2020-2022	2023
	RR (95%CI)	RR (95%CI)	RR (95%CI)	RR (95%CI)
$PM_{2.5}$	1.0007 (1.0002-1.0012)	1.0004 (0.9999-1.0009)	1.0004 (0.9998-1.0010)	1.0012 (1.0003-1.0021)
PM_{10}	1.0008 (1.0004-1.0011)	1.0008 (1.0005-1.0010)	1.0011 (1.0008-1.0014)	1.0006 (1.0003-1.0010)
O_3	1.0008 (1.0005-1.0012)	1.0007 (1.0005-1.0009)	1.0007 (1.0004-1.0010)	1.0007 (1.0003-1.0011)
SO_2	1.0158 (1.0118-1.0197)	1.0163 (1.0118-1.0208)	1.0192 (1.0140-1.0245)	1.0181 (1.0087-1.0276)
NO_2	1.0050 (1.0038-1.0063)	1.0039 (1.0028-1.0050)	1.0057 (1.0044-1.0071)	1.0026 (1.0003-1.0048)

Table S5. Two-pollutant sensitivity analyses of lag-0 associations between ambient air pollutants and conjunctivitis outpatient visits

Two-pollutant model	pollutant	Adjusted for	Lag 0 RR (95%CI)
$PM_{2.5}$ + PM_{10}	$PM_{2.5}$	PM_{10}	0.9993 (0.9988-0.9998)
$PM_{2.5}$ + PM_{10}	PM_{10}	$PM_{2.5}$	1.0011 (1.0008-1.0014)
$PM_{2.5}$ + NO_2	$PM_{2.5}$	NO_2	0.9997 (0.9993-1.0001)
$PM_{2.5}$ + NO_2	NO_2	$PM_{2.5}$	1.0049 (1.0039-1.0059)
PM_{10} + NO_2	PM_{10}	NO_2	1.0003 (1.0001-1.0005)
PM_{10} + NO_2	NO_2	PM_{10}	1.0040 (1.0031-1.0049)
